# A Zinc Oxide Nanoflower-Based Electrochemical Sensor for Trace Detection of Sunset Yellow

**DOI:** 10.3390/s17030545

**Published:** 2017-03-08

**Authors:** Yu Ya, Cuiwen Jiang, Tao Li, Jie Liao, Yegeng Fan, Yuning Wei, Feiyan Yan, Liping Xie

**Affiliations:** 1 Institute for Agricultural Product Quality Safety and Testing Technology, Guangxi Academy of Agricultural Sciences, Nanning 530007, China; yayu1026@163.com (Y.Y.); 18376767532@163.com (C.J.); 18260965051@163.com (T.L.); liao_jie1997@163.com (J.L.); fanyegeng@163.com (Y.F.); wyun2015@gxaas.net (Y.W.); yanfeiyan2014@126.com (F.Y.); 2 Quality Inspection and Test Center for Sugarcane and Its Product, China Ministry of Agriculture (Nanning), Nanning 530007, China

**Keywords:** zinc oxide nanoflower, sunset yellow, electrochemical sensor, trace detection

## Abstract

Zinc oxide nanoflower (ZnONF) was synthesized by a simple process and was used to construct a highly sensitive electrochemical sensor for the detection of sunset yellow (SY). Due to the large surface area and high accumulation efficiency of ZnONF, the ZnONF-modified carbon paste electrode (ZnONF/CPE) showed a strong enhancement effect on the electrochemical oxidation of SY. The electrochemical behaviors of SY were investigated using voltammetry with the ZnONF-based sensor. The optimized parameters included the amount of ZnONF, the accumulation time, and the pH value. Under optimal conditions, the oxidation peak current was linearly proportional to SY concentration in the range of 0.50–10 μg/L and 10–70 μg/L, while the detection limit was 0.10 μg/L (signal-to-noise ratio = 3). The proposed method was used to determine the amount of SY in soft drinks with recoveries of 97.5%–103%, and the results were in good agreement with the results obtained by high-performance liquid chromatography.

## 1. Introduction

**Scheme 1:**
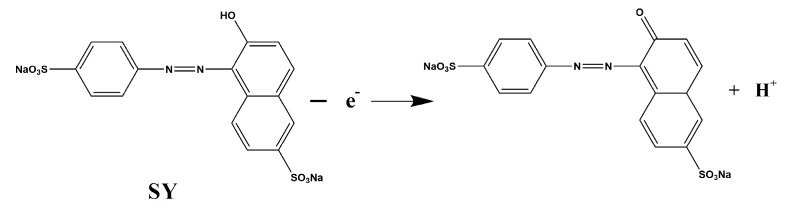
Electrochemical oxidation of sunset yellow (SY).

Sunset yellow (SY) (Scheme 1) is a synthetic dye that is commonly added into food products and soft drinks to make them more visually appealing [1]. SY is legitimately used as a food additive in China; however, if it is excessively consumed, it may cause asthma, immunosuppression, eczema, and anxiety migraines [2]. Due to the potential negative side-effects of excess SY, it is imperative to control the amount of SY in food. Consequently, a simple, rapid, and highly sensitive method for SY detection is critical to monitor SY levels. Many techniques have been utilized for the determination of SY, such as high performance liquid chromatography [3,4,5], UV-visible, fluorescence, and Raman spectrometry [6,7,8,9], and electrochemical techniques [10,11,12,13,14,15,16,17]. Recently, due to the low cost, operational simplicity, high sensitivity, and fast response, electrochemical techniques have gained attention and have exhibited promising applications in SY analysis. Based on the unique properties such as high electrical and thermal conductivity, extremely high surface area/volume ratio, high mechanical strength, and even excellent catalytic properties [18], nanomaterials have been employed as outstanding electrode modifiers for the electrochemical detection of SY. Some examples of nanomaterial-modified electrodes developed for the electrochemical determination of SY include porous carbon-modified glassy carbon electrode (GCE) [10], gold nanoparticle-modified carbon paste electrode (CPE) [11], gold nanoparticle/graphene-modified GCE [12], Au–Pd and reduced graphene oxide nanocomposite-modified GCE [13], and polypyrrole-modified oxidized single-walled carbon nanotubes-modified GCE [14].

More recently, metal oxide nanoparticles have received much attention in the field of electroanalysis due to the high surface area-to-volume ratios, biocompatibility, chemical stability, surface reaction activity, and adjustable electron transport properties [19,20]. Zinc oxide is a common metal oxide used in the electroanalysis of analytes. To date, many zinc oxide nanostructures have been utilized as electrochemical sensors, including nanoparticles, nanowires, nanorods, and nanosheets [21,22,23,24], but nanoflower-like zinc oxide is seldom used. In this paper, we describe a simple, rapid, and sensitive electrochemical method for the detection of SY using zinc oxide nanoflower (ZnONF) as sensing materials. The electrochemical behaviors of SY were studied. The result showed that the electrochemical response of SY was significantly improved at the ZnONF-modified CPE (ZnONF/CPE) when compared to the electrochemical response at the bare CPE. Finally, the ZnONF/CPE was applied to determine the amount of SY in soft drink samples, and the results were then validated by comparison with high-performance liquid chromatography.

## 2. Experimental

### 2.1. Reagents and Apparatus

5.000 mg/mL standard stock solutions of SY were obtained from the National Institute of Metrology (Beijing, China). Zinc acetate dihydrate [Zn(CH_3_COO)_2_·2H_2_O], graphite powder, paraffin oil of spectral grade, sodium hydroxide, potassium hydroxide, and phosphoric acid were purchased from Sinopharm Chemical Reagent Company (Shanghai, China). All other reagents were of analytical grade and were used as received. All aqueous solutions were prepared in deionized water. Electrochemical experiments were performed on a CHI760E electrochemical workstation (Chenhua Instrument Co. Ltd., Shanghai, China). A conventional three-electrode system was used for all electrochemical experiments. A bare or modified CPE of 3 mm diameter was the working electrode, a saturated calomel electrode was used as the reference electrode, and a platinum wire electrode was used as the auxiliary electrode. Scanning electron microscopy (SEM) characterization was conducted with a S-3400N scanning electron microscope (Hitachi Co., Tokyo, Japan). X-ray diffraction (XRD) studies were carried out using a X’Pert PRO diffractometer using Cu Kα radiation (PANalytical B.V., Almelo, Netherlands). High-performance liquid chromatography determination of SY was carried out with a Waters 2695 liquid chromatograph coupled with an UV-vis detector. Chromatographic conditions were developed employing previously described conditions [10].

### 2.2. Preparation of ZnONF

In a typical experiment, an ethanol solution of 0.1 M Zn(CH_3_COO)_2_·2H_2_O (100 mL) was prepared using mild stirring. Then, 50 mL of 1.0 M sodium hydroxide was slowly added dropwise into the above solution and the mixture was stirred for 1 h until a white suspension was obtained. The suspension was then immersed in a preheated water bath for 6 h at 65 °C. After removing the suspension from the water bath and cooling to room temperature, the resulting precipitates were centrifuged and washed with absolute ethanol and distilled water three times. Finally, the product was dried at 60 °C for 24 h to afford ZnONF.

### 2.3. Preparation of the Modified Electrode

The conventional CPE was prepared by thoroughly mixing graphite powder and paraffin oil in a ratio of 70:30 (*w*/*w*) until a uniform wetted paste was obtained. The resulting paste was firmly packed into the cavity (3.0 mm diameter) of a polytetrafluoroethylene cylindrical tube fitted with a copper piston, which served as an inner electrical contact, and the surface was polished on a smooth paper. ZnONF (10.0 mg) was added into 5.0 mL of ethanol solution, and after 30 min of ultrasonication, a stable suspension with a concentration of 2.0 mg/mL was obtained. Finally, 5.0 μL of the ZnONF suspension was coated on the CPE surface and dried in air to obtain ZnONF/CPE.

### 2.4. Analytical Procedure

Unless otherwise stated, electrochemical measurements were conducted in a conventional electrochemical cell containing 10 mL of 1/15 mol/L phosphate buffer solution (PBS) with a pH of 5.0, which contained a certain concentration of SY. After an accumulation time of 360 s under open circuit conditions, square wave voltammograms were recorded in the potential range from 0.40–1.0 V, and the oxidation peak current at 0.72 V was measured as the analytical signal for SY. Square wave voltammetry (SWV) conditions employed a frequency of 15 Hz, an amplitude of 25 mV, and a step potential of 4 mV.

## 3. Results and Discussion

### 3.1. Characterization of ZnONF and Modified Electrode

**Figure 1:**
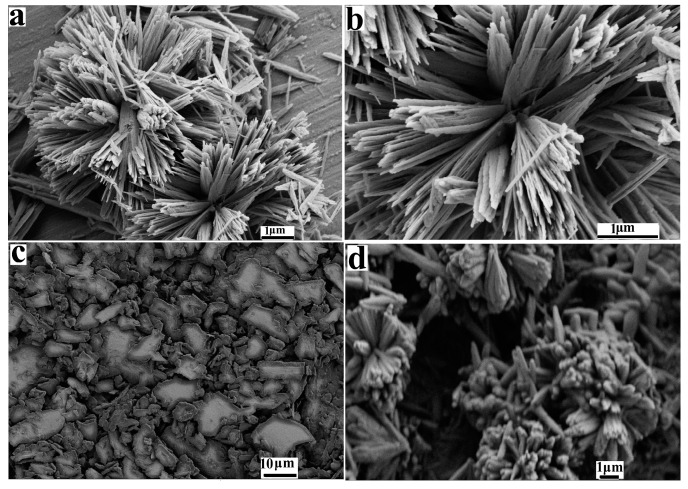
SEM images of (**a**,**b**) zinc oxide nanoflower (ZnONF); (**c**) bare carbon paste electrode (CPE); and (**d**) ZnONF-modified CPE (ZnONF/CPE).

Figure 1a,b shows the typical SEM images of the as-synthesized ZnONF. As observed in the SEM images, the ZnONF nanostructures present a flower-like shape consisting of many whisker-like nanopetals. Undoubtedly, such a structure is highly beneficial for maintaining a high surface area and numerous adsorption sites on the electrode for use in the electrochemical detection of SY. The surface morphologies of the bare CPE and the ZnONF/CPE were also characterized by SEM (Figure 1 c,d). As indicated by the SEM images, the surface of bare CPE is smooth and with many small gaps. After ZnONF was added to the surface of CPE, the ZnONFs were distributed on the surface of the electrode with a well-defined flower-like shape, indicating that the ZnONFs were successfully modified on the CPE surface.

**Figure 2:**
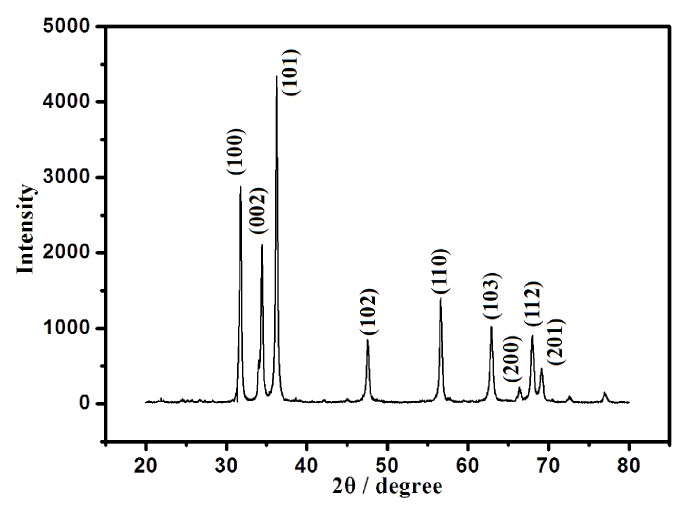
XRD pattern of ZnONF.

The crystalline structure of ZnONF was characterized by XRD, as shown in Figure 2. Nine peaks of the prepared sample were present corresponding to (100), (002), (101), (102), (110), (103), (200), (112), and (201) crystal face, which agree well with the standard XRD pattern of ZnO (JCPDS Card No. 36-1451) [25,26]. The strong and narrow diffraction peaks indicate that the prepared ZnONF is well crystallized. No characteristic peaks from impurities were detected, indicating that the product is rather pure.

### 3.2. Cyclic Voltammetric Behavior of SY

**Figure 3:**
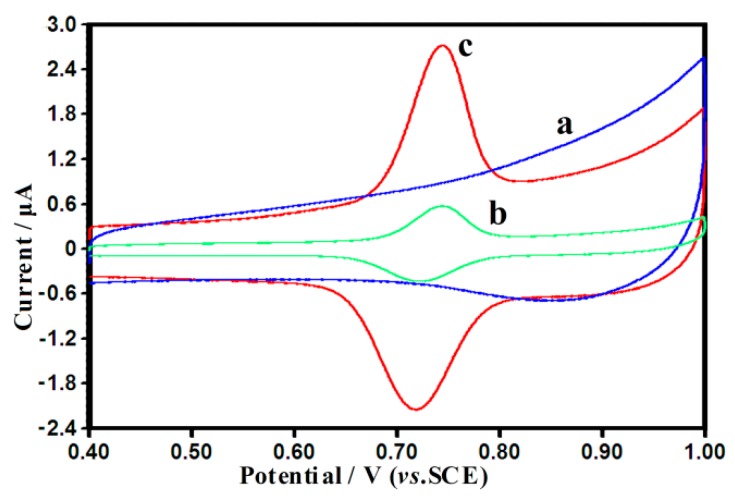
Cyclic voltammograms of (a) the ZnONF/CPE in pH 5.0 phosphate buffer solution (PBS) without SY; (b) the 40 μg/L SY in pH 5.0 PBS at the bare CPE; and (c) the ZnONF/CPE.

The electrochemical behaviors of SY at different electrodes were investigated using cyclic voltammetry (CV) in PBS (1/15 mol/L, pH = 5.0). As shown in Figure 3, there were no redox peaks in the ZnONF/CPE in PBS without SY (curve a). When SY was added into the PBS, a pair of reversible redox peaks were observed in the cyclic voltammograms obtained at the bare CPE with a poor current response (curve b). In contrast, the redox peaks of SY at the ZnONF/CPE were greatly enhanced (curve c), indicating that ZnONF can provide superior electrocatalytic activity to SY. This may be ascribed to the good adsorption ability and large surface area of ZnONF, which increase the loading amount of SY onto the surface of the modified electrode. Therefore, a remarkable increase of electrochemical signal was observed at the ZnONF/CPE.

### 3.3. Influence of Scan Rate

**Figure 4:**
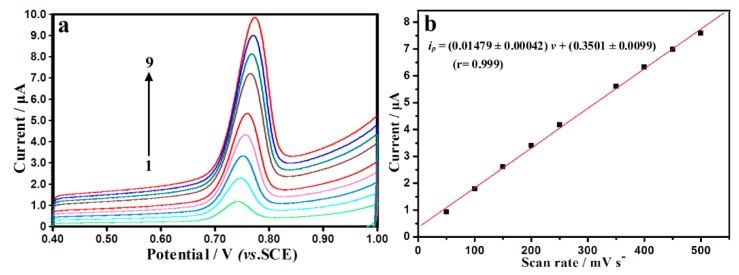
(**a**) Linear sweep voltammograms of 40 μg/L SY at the ZnONF/CPE in pH 5.0 PBS with different scan rates (from 1 to 9: 50, 100, 150, 200, 250, 350, 400, 450, and 500 mV/s); (**b**) Linear relationship between the current response and the scan rate.

The influence of scan rates on the oxidation current response of SY was studied by linear sweep voltammetry in the range of 50–500 mV/s. Figure 4 demonstrates that the current responses increased linearly with scan rate, and consequently, the linear equation can be expressed as follows: *i_p_* (μA) = (0.01479 ± 0.00042) *v* (mV/s) + (0.3501 ± 0.0099) (r = 0.999). These results show that the electrochemical oxidation of SY at the ZnONF/CPE is an adsorption-controlled process.

### 3.4. Optimization of Detection Variables

**Figure 5:**
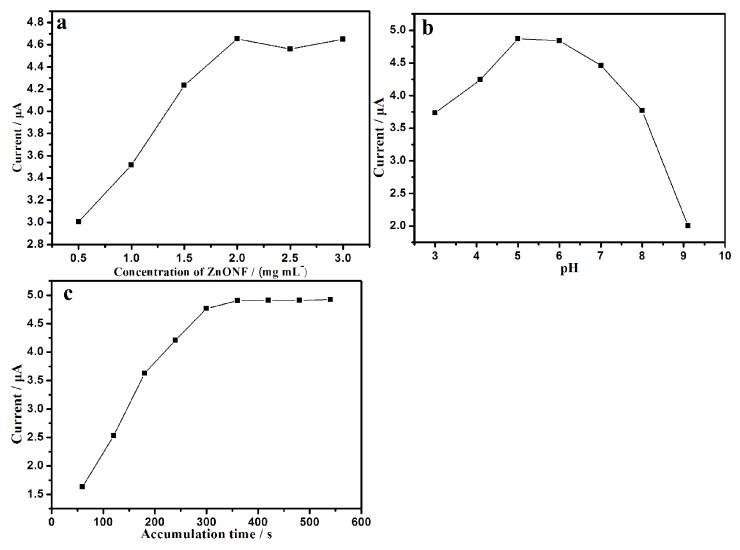
The effect of the (**a**) concentration of ZnONF; (**b**) pH; and (**c**) accumulation time on the current response of 40 μg/L SY.

The effect of the concentration of ZnONF on current response of SY was investigated, and the results are shown in Figure 5a. As the concentration of ZnONF increased from 0.50 to 2.0 mg/mL, the current response of SY increased greatly. When the amount of ZnONF was increased, increasing amounts of SY were adsorbed on the surface of ZnONF/CPE, which resulted in a remarkable current response enhancement. As the current response of SY changed slightly when the concentration of ZnONF was greater than 2.0 mg/mL, the optimal concentration for ZnONF was determined to be 2.0 mg/mL.

Figure 5b displays the effect of pH on the current response of SY. When the pH was increased from 2.0 to 5.0, the current response likewise increased, and a plateau appeared between pH 5.0 to 6.0. For pH values greater than 6.0, the current response decreased as pH further increased. Therefore, 1/15 mol/L PBS with pH of 5.0 was used as the electrolyte in this work.

The accumulation efficiency of ZnONF/CPE to SY was studied under open-circuit conditions. As Figure 5c illustrates, the current response of SY was dramatically improved by increasing the accumulation time from 0 to 360 s, indicating that accumulation is effective in improving the detection sensitivity. When the accumulation time was further increased beyond 360 s, the current response was almost constant. This may be due to saturation of the amount of SY adsorbed on the surface of ZnONF/CPE. Thus, 360 s was selected as the optimal accumulation time for the determination of SY.

### 3.5. Analytical Properties

**Figure 6:**
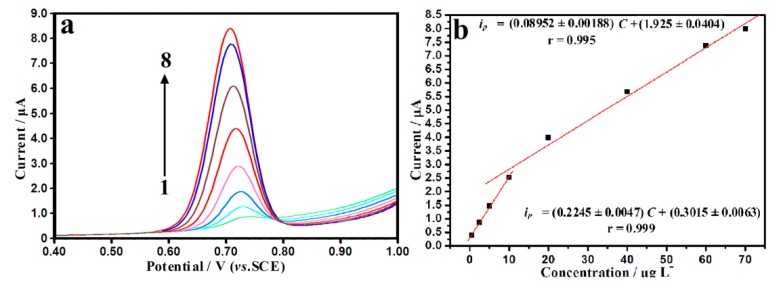
(**a**) Square wave voltammetry (SWV) curves of SY at the ZnONF/CPE in pH 5.0 PBS with various concentrations. Curves 1 to 8 correspond to 0.50, 2.50, 5.0, 10, 20, 40, 60, and 70 μg/L of SY, respectively; (**b**) The current response as a function of the SY concentration.

The linear range and detection limit for SY were examined using SWV under the optimized conditions. As can be seen from Figure 6, the current response of SY is linearly related to SY concentration over a range of 0.50–10 μg/L and 10–70 μg/L. The linear regression equation can be expressed as follows: *i_p_* (μA) = (0.2245 ± 0.0047) *C* (μg/L) + (0.3015 ± 0.0063) and *i_p_* (μA) = (0.08952 ± 0.00188) *C* (μg/L) + (1.925 ± 0.0404) with the correlation coefficients of 0.999 and 0.995. The detection limit was estimated to be 0.10 μg/L (signal-to-noise ratio = 3). The comparison of ZnONF/CPE with other types of material modified electrodes for SY determination are listed in Table 1. As indicated in Table 1, the ZnONF/CPE exhibited high sensitivity and low detection toward the electrochemical determination of SY.

The reproducibility of the ZnONF/CPE was examined using a PBS containing 20 μg/L SY, and the relative standard deviation (RSD) of the current response was determined to be 4.22% by using six independently-prepared ZnONF/CPE. The reproducibility of one ZnONF/CPE was estimated by recording the responses of 20 μg/L SY in 11 successive measurements, and the RSD was calculated to be 5.31%. The sample-to-sample reproducibility was also examined, and the RSD was 2.57% for three time measurement. The stability of the ZnONF/CPE was investigated after long-term storage in a refrigerator at 4 °C for one week, and 93.7% of the original oxidation peak current was retained. From these results, it is evident that the ZnONF/CPE exhibits good reproducibility and stability.

The influence of various foreign species on the determination of 10 μg/L SY was investigated by SWV using the above-optimized conditions. For this work, the tolerance limit was defined as the molar ratio of foreign species/SY that caused the ±5.0% change in the current response of SY. The result showed that 500-fold of K^+^, Na^+^, Ca^2+^, Mg^2+^, Cu^2+^, Zn^2+^, Fe^3+^, sucrose, glucose, citric acid, and vitamin C; 50-fold of Sudan red, ponceau 4R, allura red, and amaranth; and 20-fold of tartrazine did not interfere in the determination of SY. These results demonstrate that ZnONF/CPE has good selectivity for SY.

The analytical applicability of the ZnONF/CPE for the determination of SY was examined using the SWV method under optimized experimental conditions. Ten microliters of the soft drink sample was added to 10 mL of PBS and used for the determination of SY. A standard addition method was adopted to estimate the accuracy of the proposed method. The results are given in Table 2. The recoveries of the standards added ranged from 97.5% to 103%. The concentration of SY was also detected using HPLC, and the results obtained by HPLC and the proposed method were in good agreement, indicating that the proposed method is reliable.

## 4. Conclusions

In this work, we have designed an ultrasensitive electrochemical sensor for the detection of SY based on the ZnONF/CPE. The ZnONF with unique nano-structure exhibited a large surface area and outstanding binding capacity, which greatly improved the electrochemical oxidation signals of SY. The fabricated sensing system demonstrated advantages including high sensitivity, good selectivity, simple fabrication, and low cost, indicating that the ZnONF/CPE could be employed as a highly promising tool for electrochemical sensing.

## Figures and Tables

**Table 1:** Comparison of different modified electrodes for SY determination. GCE: glassy carbon electrode.

Electrode	Linearity Range (μg/L)	Detection Limit (μg/L)	Reference
Porous carbon-modified GCE	2.5–500	1.4	[10]
Gold nanoparticles-modified GCE	23–723	0.90	[11]
Nanoparticles/graphene-modified GCE	0.9–49,372	0.90	[12]
Au-Pd and reduced graphene oxide Nanocomposites-modified GCE	310–150,048	0.68	[13]
Polypyrrole-modified oxidized single-walled Carbon Nanotubes-modified GCE	2.3–452	0.32	[14]
Poly (l-cysteine)-modified GCE	3.6–317	1.8	[15]
Bismuth film-modified GCE	4.4–87	1.0	[16]
Functionalized montmorillonite-modified CPE	1.1–90	0.32	[17]
ZnONF/CPE	0.50–10 and 10–70	0.10	This work

**Table 2:** Determination and recovery of SY in soft drink sample.

Original (mg/L)	Added (mg/L)	Found (mg/L)	Recovery (%)	By HPLC (mg/L)	Relative Error (%)
	20.00	33.07	97.5	31.42	−4.99
13.57	30.00	42.25	95.6	45.05	6.63
	40.00	54.71	103	53.12	−2.91
